# Correction: Combining lenalidomide with IL-2 family of cytokines enhances activating receptor and perforin/granzyme expression in NK cells

**DOI:** 10.1371/journal.pone.0348020

**Published:** 2026-04-22

**Authors:** Alexandra Calescibetta, Robert Dalton, Nicole Fortenbery, Grace Ward, Sean Christiansen, Xianghong Chen, Pingyan Cheng, Tiffany Razabdouski, Annelise J. Glode, Nhan Tu, Thu Le Trinh, Jinghong Liu, Kenneth L. Wright, Sheng Wei, Erika Adriana Eksioglu

Fig 2 is showing the data for Fig 6 in repeat. Data for Fig 2 is missing. Please view Fig 2 here.

**Fig 2:**
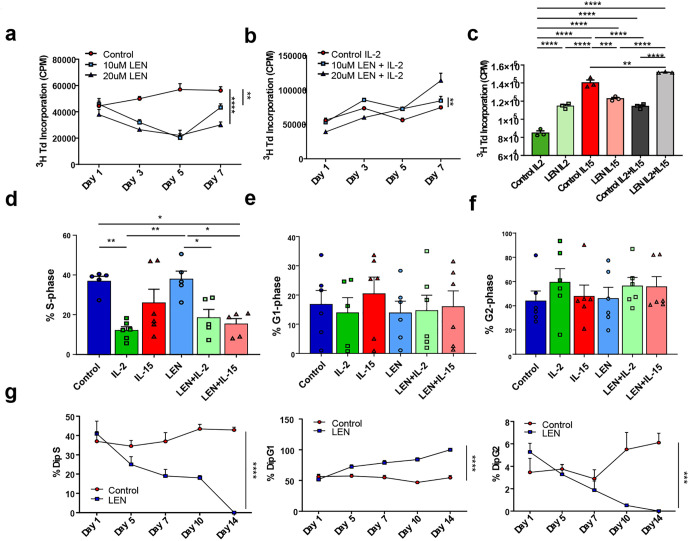
Combination with IL-2 with Lenalidomide’s effects on NK cell cycle. Proliferation of YT cells measured by overnight tritium (3H) incorporation (1.0 μCi per well) after culture for 1,3,5 or 7 days in the presence of 10 or 20μM lenalidomide (LEN) in the (a) absence or (b) presence of human recombinant (hr) IL-2 (100U/mL) or (c) combined results of YT proliferation at 7 days for coculture of lenalidomide with IL-2 and/or IL-15. Results expressed as mean counts per minute (cpm) of triplicate wells for each experiment, and a total of n = 3 biological replicates. Cell cycle analysis of isolated primary NK cells (n = 6 healthy donor biological replicates), treated as indicated, measured via flow cytometric analysis of propidium iodide (PI) staining and expressed as percent of cells in **(d)** S, **(e)** G1, or **(f)** G2 phase. **g)** Cell cycle analysis of YT cells cultured with 20μM LEN (for up to 14 days) measured via flow cytometric analysis of propidium iodide (PI) staining and expressed as percent of cells in S, G1 and G2 phase. Error bars represent the SEM and were analyzed by 2way ANOVA. P values are shown as asterisk: * P ≤ 0.05, ** P ≤ 0.01, *** P ≤ 0.001, **** P ≤ 0.0001 as summarized in Graphpad Prism.
